# High acceptability, feasibility and sustainability of a direct‐to‐pharmacy differentiated PrEP delivery model in public health HIV clinics in Kenya: perspectives of PrEP clients and healthcare providers

**DOI:** 10.1002/jia2.26442

**Published:** 2025-07-07

**Authors:** Emmah Owidi, Kenneth Ngure, Vallery Ogello, Njeri Wairimu, Lydia Etyang’, Winnie Waituika, Margaret Mwangi, Dominic Mwangi, Simon Maina, Elizabeth Irungu, Catherine Kiptinness, Nelly Mugo, Kenneth Mugwanya, for the Efficiency Study Team

**Affiliations:** ^1^ Partners in Health and Research Development Center for Clinical Research, Kenya Medical Research Institute Nairobi Kenya; ^2^ School of Public Health Jomo Kenyatta University of Agriculture and Technology Nairobi Kenya; ^3^ Department of Global Health University of Washington Seattle Washington USA; ^4^ Jhpiego Nairobi Kenya; ^5^ Center for Clinical Research Kenya Medical Research Institute Nairobi Kenya; ^6^ Department of Epidemiology University of Washington Seattle Washington USA

**Keywords:** direct‐to‐pharmacy, public HIV clinics, implementation science, Kenya, differentiated service delivery, pre‐exposure prophylaxis

## Abstract

**Introduction:**

High client opportunity costs and a burdened healthcare system limit oral pre‐exposure prophylaxis (PrEP) delivery in Kenyan public HIV clinics. We conducted a qualitative study among PrEP clients and providers to understand the acceptability, feasibility and willingness to implement a client‐centred, differentiated direct‐to‐pharmacy (DTP) PrEP refill visits intervention aimed at improving the efficiency of PrEP implementation in real‐world clinics.

**Methods:**

From March 2021 to March 2022, we conducted in‐depth interviews with clients and healthcare providers participating in an individual facility pharmacy‐based PrEP delivery model for PrEP refills among clients in the continuation phase at two public HIV clinics in central Kenya. The core components of the DTP model included directed‐to‐PrEP pharmacy refill visits conducted by facility pharmacy staff and client HIV self‐testing (HIVST) while waiting for services at the pharmacy. We used semi‐structured interview guides informed by the Consolidated Framework for Implementation Research (CFIR). We analysed data using thematic content analysis and organised findings by CFIR constructs.

**Results:**

We interviewed 20 PrEP clients and 20 healthcare providers. PrEP clients included 15 females and had a median age of 39 years (interquartile range [IQR]: 33–48). Providers included 13 females, had a median age of 32 years (IQR: 30–41), and included 10 HIV counsellors, 5 pharmacy and 3 clinical providers. Both providers and clients reported high satisfaction with DTP PrEP refill visits derived from improved clinic flow and quality of service. Among clients, shorter waiting times and less movement between multiple clinic rooms reduced delays, improved privacy and reduced stigma associated with visiting HIV clinics. Furthermore, shorter waiting times and infrequent clinic visits reduced loss of working hours and income among clients, motivating PrEP continuation. Providers reported improved clinic flow, reduced work burden among non‐pharmacy providers, improved knowledge and ease of implementing DTP refill visits. However, providers expressed concerns about the potential loss of roles among HIV counsellors and the shifting of workload burden to pharmacy providers.

**Conclusions:**

Differentiated DTP refill visits with HIVST were highly acceptable and feasible among PrEP clients and providers. Context‐specific modifications and scale‐up of the intervention could improve the efficiency of PrEP delivery within public HIV clinics in Kenya and similar settings.

## 1 INTRODUCTION

Oral HIV pre‐exposure‐prophylaxis (PrEP) is a highly effective and safe HIV prevention strategy among populations at substantial risk of HIV acquisition [1, 2]. PrEP uptake, adherence and continuation have been sub‐optimal among populations that could benefit from PrEP in high HIV‐burden, resource‐limited regions [3, 4, 5]. In eastern and southern African countries, inadequate staffing and infrastructure, long waiting times and frequent refill visits are major health system barriers that hinder efficient PrEP delivery and access in public HIV clinics [6, 7]. These barriers exacerbate already burdened healthcare systems and increase clients’ opportunity costs [8, 9]. Eliminating clinic‐based barriers can reduce opportunity costs and HIV‐clinic‐associated stigma, contributing to higher PrEP uptake and continuation, particularly among sub‐populations that show high initial interest in PrEP but experience challenges in adherence and continuation [10, 11].

Studies in sub‐Saharan Africa (SSA) have revealed distinct patterns of PrEP uptake and continuation among priority sub‐populations such as adolescent girls and young women (AGYW), heterosexual men and women, and individuals in HIV‐serodifferent partnerships [12, 13]. Early PrEP discontinuation has been reported among individuals with significant ongoing HIV risk, including AGYW and people with partners of unknown HIV status or multiple sexual partners [11, 12, 14]. Gender differences in health‐seeking behaviours, low HIV and PrEP knowledge, and low HIV risk perceptions among young people have been linked to early discontinuation [14, 15, 16]. Providing options through differentiated service delivery (DSD) approaches could cater to differences in individual needs and preferences [5, 17]. Differentiated service strategies simplify care for clients, are tailored to individual needs and preferences, and maximise health system impacts, efficiency and cost‐effectiveness [18]. The World Health Organization (WHO) recommends DSD to eliminate PrEP access barriers and to enhance its uptake, continuation and effective use [18]. Differentiated care interventions can improve the acceptability and effectiveness of PrEP services, and support PrEP scale‐up and implementation [18, 19].

Adapting the “where,” “who,” “when” and “what” components of DSD through service decentralisation, task‐sharing, multi‐month dispensing, and separated clinical consultation and refill‐only visits, has optimised HIV care and treatment programmes, yielding improved clients’ adherence, retention and viral suppression outcomes [20, 21, 22, 23]. Similar differentiated interventions have been applied within PrEP delivery in Kenya and other SSA countries, demonstrating high levels of acceptability and feasibility [24, 25, 26, 27, 28]. Few interventions have focused on improving service efficiency and quality in public health HIV clinics, despite these being among the largest PrEP delivery locations in SSA [29, 30]. Our study sought to demonstrate the feasibility, acceptability and sustainability of direct‐to‐pharmacy (DTP) PrEP refill visits with HIV self‐testing (HIVST), an individual facility‐based differentiated PrEP care model, within public health HIV clinics in central Kenya.

## 2 METHODS

### 2.1 Study design and approach

The Efficiency Study (ClinicalTrials.gov: NCT04424524) was a prospective pilot implementation evaluation of a client‐centred, differentiated care intervention aimed at improving the efficiency of PrEP delivery in Kenyan public HIV clinics. The study's overall objective was to demonstrate the feasibility and acceptability of DTP PrEP follow‐up visits among PrEP clients in the continuation phase, with the key expected outcomes including greater efficiency, reduced workload, diminished client burden, and improved service and client outcomes. The study design and approach have been previously described [31]. Four HIV clinics participated in this pilot study, two implementing a differentiated pharmacy‐based follow‐up PrEP care pathway (DTP), and two a standard PrEP client flow without any change (control). The clinics were purposively selected from 12 facilities that implemented PrEP in the Partners Scale‐Up Project [32], based on willingness to implement DTP, high clinic volume and space availability.

In the intervention arm, clients accessed 3‐monthly DTP PrEP refill visits. At the initiation visit, clients were informed they would receive PrEP services directly at the pharmacy at their next follow‐up visit. At follow‐up, a PrEP clients’ navigator retrieved client files and directed clients to the pharmacy, where a pharmacy provider conducted a rapid risk assessment (for ongoing HIV risk, adherence and side effects), and PrEP dispensing. Clients conducted HIVST at a private place at the pharmacy while waiting for PrEP refills and had the opportunity to access clinical review from a clinician on request or if they had an indication. Under the standard client flow, clients accessed 3‐monthly PrEP refills with clinical review and follow‐up HIV testing as per Kenya's HIV prevention and treatment guidelines [33]. Clients had numerous stopovers for services, including HIV risk assessment and testing by an HIV counsellor, clinical review and PrEP prescription by a clinician, and PrEP dispensing by a pharmacy provider.

Overall, 746 PrEP clients (380 DTP; 366 control) were enrolled in the study and followed up for 6 months. The primary quantitative findings of this study have been previously described [31]. This paper presents the results of an in‐depth qualitative assessment conducted among a sub‐set of PrEP clients and healthcare providers in the intervention arm, who had experience with the DTP model, to understand their experiences and perceptions about the intervention.

### 2.2 Study population and setting

We conducted this study in two healthcare facilities: Facility A (Level 4) and Facility B (Level 5) in central Kenya, with county population‐level HIV prevalence rates of up to 2.7% [34]. We enrolled PrEP clients and providers to participate in in‐depth interviews. Eligible clients and providers were aged ≥ 18 years and were able and willing to provide written consent. Additionally, clients were not living with HIV, based on negative HIV tests per Kenya's HIV guidelines and were currently or previously accessing PrEP at the intervention clinics. Clients were purposively sampled by sex, while providers were sampled by cadres (HIV counsellors, pharmacy providers, clinicians, nurses) and experience in implementing DTP refill visits in the intervention clinics. Eligible clients were informed about the study by clinic staff, and the contact information of those willing to participate was shared with the researchers for interview scheduling. Providers were recruited by the researchers during in‐person facility visits. Our sample size was guided by the principle of theme saturation, where we stopped interviews once no new information emerged [35].

### 2.3 Data collection

We conducted in‐depth interviews between March 2021 and March 2022 to obtain an in‐depth understanding of participants’ experiences and perceptions about the DTP intervention [36]. All interviews were conducted in‐person by three Bachelors‐level female social scientists in private rooms at the HIV clinics in the participants’ preferred language (English or Kiswahili), each lasting approximately 1 hour. We used separate semi‐structured interview guides for clients and providers informed by the Consolidated Framework for Implementation Research (CFIR). We used CFIR during data collection and analysis to identify the relevant contextual barriers and facilitators that influenced the implementation of DTP refill visits to guide the design of tailored implementation strategies [37]. Relevant CFIR constructs were applied to explore the outcomes of the DTP refill visits, including their acceptability, feasibility and sustainability within our setting.

### 2.4 Data management and analysis

Interviews were audio‐recorded, transcribed and translated into English. After each interview, the interviewers summarised key points in a debriefing report. Interview data was analysed inductively and deductively using thematic content analysis [38]. All analyses were performed on de‐identified data using Dedoose Version 9.2.007 (Sociocultural Research Consultants LLC, Los Angeles, CA, 2021) [39]. First, EO reviewed all the transcripts, generated key themes and developed separate codebooks for clients and providers based on the CFIR framework and the interview guides. Authors EO, VO and NW selected a subset (15%; *n* = 3) of the client and provider interviews and double‐coded them for consistency. The authors reviewed the codes and resolved discrepancies over weekly meetings until an agreement was reached. EO and VO independently coded the remaining transcripts and grouped the emerging themes from the interviews. Once coding was complete, the authors summarised key themes and compared findings from clients and providers.

### 2.5 Ethical statement

The University of Washington Human Subjects Ethical Committee and the Scientific and Ethics Review Unit of the Kenya Medical Research Institute approved this study. All participants provided individual written informed consent before study participation.

## 3 RESULTS

### 3.1 Participant characteristics

We interviewed 20 PrEP clients and 20 healthcare providers. Among clients, 75% were female (*n* = 15), the median age was 39 years (IQR: 33–48) and PrEP use experience was 6 months (IQR: 5–26). Of the providers, 65% (*n* = 13) were female, the median age was 32 years (IQR: 30–41) and PrEP delivery experience was 42 months (IQR: 12–48), as outlined in Table 1.

**Table 1:** Participant demographic characteristics

	PrEP clients (*N* = 20)			PrEP providers (*N* = 20)		
	All	Facility A (*n* = 10)	Facility B (*n* = 10)	All	Facility A (*n* = 10)	Facility B (*n* = 10)
**Sex**						
Female	15 (75%)	7 (70%)	8 (80%)	13 (65%)	7 (70%)	6 (60%)
Male	5 (25%)	3 (30%)	2 (20%)	7 (35%)	3 (30%)	4 (40%)
**Age**						
Mean (SD)	39 (10)	43 (7)	35 (10)	37 (12)	34 (8)	39 (15)
Median [IQR]	39 [33−48]	43 [38−50]	34 [27−39]	32 [30−41]	32 [29−40]	34 [30−40]
**Relationship type**						
HIV‐serodifferent	17 (85%)	10 (100%)	7 (70%)	−	−	−
Unknown partner status	3 (15%)	0 (0%)	3 (30%)	−	−	−
**Current position**						
Clinic manager (Nurse)	−	−	−	1 (5%)	0 (0%)	1 (10%)
Clinical officer	−	−	−	1 (5%)	1 (10%)	0 (0%)
Nurse	−	−	−	1 (5%)	0 (0%)	1 (10%)
Health records and information officer	−	−	−	1 (5%)	1 (10.0%)	0 (0%)
HIV counsellor	−	−	−	10 (50%)	6 (60%)	4 (40%)
Pharmacy provider	−	−	−	5 (25%)	1 (10%)	4 (40%)
Social worker	−	−	−	1 (5%)	1 (10.0%)	0 (0%)
**Participation in PrEP delivery (months)**						
Mean (SD)	−	−	−	32 (19)	36.8 (16.2)	26 (20)
Median [IQR]	−	−	−	42 [12−48]	48 [27−48]	24 [7−48]
**Duration of PrEP use (months)**						
Mean (SD)	18 (23)	17 (17)	20 (29)	−	−	−
Median [IQR]	6 [5−26]	7 [5−31]	6 [4−23]	−	−	−

Abbreviations: IQR, inter‐quartile range; PrEP, pre‐exposure prophylaxis; SD, standard deviation.

Clients and providers reported that DTP PrEP refill visits resulted in improved service efficiency, quality and satisfaction attributed to reduced waiting times, less movement between clinic rooms and infrequent clinic visits. Table 2 summarises the key determinants and outcomes of DTP refill visits related to their acceptability, feasibility and sustainment.

**Table 2:** Perceived determinants and outcomes of DTP PrEP refill visits by CFIR constructs

CFIR domain/construct	DTP PrEP refill visits determinants and outcomes	Beneficiary/ affected
**Innovation**		
Relative advantage	**Waiting time**: Reduced waiting time and improved time efficiency.	Clients
		Providers
	**Client flow**: Reduced queuing and movement between clinic rooms, smooth clinic flow, reduced congestion.	Clients
		Providers
		Health system
	**Workload**: General reduced burden among clinic staff; concerns of potential workload shift to pharmacy providers.	Providers
Adaptability	**Clinic modifications**: Pre‐scheduled PrEP refill visit dates and differentiated PrEP delivery spaces.	Clients
Complexity	**Low complexity**: Ease of implementing PrEP refills.	Providers
**Outer setting**		
Local attitudes	**Service quality and satisfaction**: Improved service quality, satisfaction, convenience and comfort.	Clients
		Providers
	**Privacy and HIV stigma**: Improved privacy and reduced risk of exposure to HIV‐clinic‐associated stigma.	Clients
**Inner setting**		
Work infrastructure	**Staffing organisation**: Adequate clinic staffing overall, but shortage of pharmacy and HIV counselling providers.	Providers
		Health system
Physical/information technology infrastructure	**Clinic infrastructure**: Adequate overall but inadequate rooms and electronic medical records (EMR) systems.	Providers
		Health system
Recipient‐centredness	**Differentiated refill visits**: Prioritising and fast‐tracking PrEP refill clients, client navigation.	Clients
Deliverer‐centredness	**Task‐sharing**: Improved teamwork and flexibility in PrEP service delivery.	Providers
Learning‐centredness	**Knowledge and skills**: Increased empowerment and knowledge in HIV prevention and PrEP delivery skills.	Clients
		Providers
		Health system
Compatibility	**Ease of adoption**: Few changes to routine operations, addressed service challenges and staff shortage.	Health system
		Providers
Relative priority and mission alignment	**PrEP prioritisation**: Facilitated prioritisation of PrEP services and achievement of HIV prevention targets.	Health system
Access to knowledge and information	**Provider training**: Adequate PrEP delivery training facilitated intervention implementation and ownership.	Providers
**Individuals**		
**Clients’ needs**	**Opportunity costs**: Reduced time and costs of travel to clinics, less disruption of clients’ schedules and less worry over missing important activities.	Clients
**Capability**	**Self‐efficacy**: Increased skills and confidence in implementing DTP refill visits and PrEP delivery.	Providers
**Motivation**	**Future willingness**: General confidence and willingness to participate in DTP refill visits.	Clients
		Providers

Abbreviations: CFIR, Consolidated Framework for Implementation Research; DTP, direct‐to‐pharmacy; PrEP, pre‐exposure prophylaxis.

### 3.2 Innovation domain

#### 3.2.1 Innovation relative advantage

##### 3.2.1.1 Improved waiting time

PrEP clients and providers preferred DTP refill visits due to short waiting times and few delays in HIV clinics. Short waiting times were attributed to clients seeing few (2−3) providers, less movement between clinic rooms, ease and convenience of HIVST, and prioritisation of PrEP clients during refill visits. The short waiting times motivated clients to continue PrEP:

*“[DTP] has really given me the morale [motivation] of taking the drugs because you spend less time in the hospital. Also, you are dispensed drugs for three months, not like before when it used to be for one month only.”* (Female Client, Age 33, Facility A).

Short waiting times were less preferred among older clients, particularly males (≥ 45 years) in HIV‐serodifferent relationships who reported prioritising their health over waiting time and perceived that seeing few providers resulted in inadequate counselling and clinical assessments. HIV counsellors and triage nurses shared concerns about possible missed opportunities for clinical assessments, adherence counselling and psychosocial support for HIV‐serodifferent couples.

##### 3.2.1.2 Improved client flow

Providers perceived DTP refill visits to improve client flow, attributed to less queuing and movement of clients within HIV clinics and fewer PrEP delivery steps. This resulted in faster service delivery, reduced clinic congestion and freed up providers to perform other roles:

*“[DTP] has improved the workflow; you can see more patients, we have more time for other things, you are spending less time on one client…Before, [clients] used to go to the clinician, the social worker…So, they are free to attend to other more needy clients.”* (Male Pharmacist, Facility B).

##### 3.2.1.3 Reduced workload burden

DTP refill visits were perceived to be less burdensome among non‐pharmacy providers due to the fewer service delivery steps and less interaction with PrEP clients compared to standard client flow. Clients’ use of HIVST also reduced workload from provider‐conducted HIV testing:

*“[Workload] has changed significantly because you can imagine all the people who normally come to the HIV clinic for PrEP coming to the HIV counsellor for HIV testing. Now…we give them self‐test and they go and test themselves”*… (Male HIV Counsellor, Facility A).

Providers across all cadres reported concerns of increased pharmacy burden, attributed to pharmacy staff shortage, and added PrEP documentation and counselling roles. However, pharmacy providers reported high to moderate workload, relative to daily changes in clinic volume:

*“[Workload] is not something that we can't handle; it's just adequate, it's not overburdening, it's okay. I cannot say that the workload is too much.”* (Female Pharmacist, Facility B).

#### 3.2.2 Innovation adaptability

Clinics reported modifications to accommodate DTP refill visits, including pre‐scheduling PrEP clients’ refill visits, to manage the number of PrEP clients visiting the clinics per day. This ensured a manageable provider workload and facilitated clinic flow planning. Changes were also made to service delivery areas to ease clinic flow and enhance the privacy and comfort of PrEP clients:

*“[PrEP clients] are served by the same pharmacist but from a different setting because HIV clients sit outside, they queue outside the pharmacy…but PrEP clients go inside the pharmacy, there is a hall there for them to sit on.”* (Female Clinic Manager, Facility B).

#### 3.2.3 Innovation complexity

Performing DTP refill visits was perceived as easy and uncomplicated, “smooth,” “straightforward” and “easy to learn,” attributed to the few steps and procedures involved, use of HIVST, reduced workload and improved provider PrEP delivery skills:

*“[DTP] is easy because you are empowered, you understand all the process. So, when the client comes, you are able to serve them as it should be and refer them where it's appropriate.”* (Female HIV Counsellor, Facility B).

### 3.3 Outer setting domain

#### 3.3.1 Community attitudes

##### 3.3.1.1 Improved service quality and satisfaction

PrEP clients reported high service quality and satisfaction with DTP refill visits, attributed to good interaction with providers, receiving adequate counselling, and comfort and convenience of short waiting times, fewer queues and seeing few providers, which motivated PrEP continuation:

*“I always feel comfortable. I feel so happy in my heart because I see that [providers] are so concerned with me…I told my husband that we always have an easy time here. We are always done in one minute, and we leave.”* (Female Client, Age 40, Facility B).

Providers and clients also perceived improvement in service quality and satisfaction related to reduced clients’ complaints about lengthy clinic waiting times:

*“There was a time we [clients] were so many…and everyone was complaining about going to work. Now in your mind, you are just like, “…I will come back when there are no [fewer] people”…But now…there are a few people.”* (Female Client, Age 33, Facility A).

Service dissatisfaction among clients was related to concerns about inadequate counselling due to the short time spent with providers, particularly among older male clients (≥ 45 years). However, younger female clients (≤ 40 years) reported that they could request counselling if they had a problem. Two clients reported experiencing long queues at the pharmacy. This concern was shared by HIV counselling and pharmacy providers, who worried about longer waiting times at the pharmacy during high‐volume clinic days, which potentially affected service quality:

*“At times, the patients are so many that you get overwhelmed; so it affects [DTP] because when you are tired, the type of services you offer to patients in the morning is not the same as what you will do in the afternoon”*… (Female Pharmaceutical Technologist, Facility A).

##### 3.3.1.2 Improved privacy and reduced HIV‐clinic‐associated stigma

DTP refill visits were perceived to improve clients’ privacy and reduce the stigma associated with receiving care at HIV clinics. Among younger male and female clients (≤ 40 years), spending less time at the clinics reduced the risk of being seen by familiar people and seeing few providers reduced the embarrassment of sharing personal issues with multiple providers. Use of HIVST and client navigation also expedited clients’ access to services, reducing privacy and stigma concerns:

*“One thing I love [about DTP] is privacy; if I test myself and go directly to the pharmacy, they will give me my drugs, and I will put them in my bag, then I leave. So, before other people think or see me there, I have already gone.”* (Male Client, Age 40, Facility A).

### 3.4 Inner setting domain

#### 3.4.1 Staffing organisation

Clinics reported the availability of adequate staffing for implementing DTP refill visits. Providers performed different roles during PrEP initiation or refill visits, including training and assisting clients to perform HIVST and providing PrEP and HIV prevention counselling (HIV/adherence counsellors), PrEP prescribing and dispensing (clinicians/pharmacists) and client scheduling (health records and information officers [HRIOs]). However, occasions of shortages were reported among HIV counselling and pharmacy providers:

*“In [facility A], we only have one pharmacist…who is dispensing [medication]. So, the workload is too much bearing in mind in [facility A], we have more than eighty patients getting into the clinic every day.”* (Female Pharmaceutical Technologist, Facility A).

#### 3.4.2 Physical and information technology infrastructure

Clinics reported adequate infrastructure and resources for PrEP delivery during DTP refill visits. However, there were inadequate private spaces for HIVST and pharmacy procedures, which providers addressed by utilising alternative clinic rooms. Shortages of commodities, including HIVST kits, during the COVID‐19 pandemic were also reported. Electronic medical records (EMR) systems facilitated the documentation of clients’ records. However, in one clinic, PrEP clients’ records were not integrated into the EMR, and the manual records were inconvenient for providers:

*“There hasn't been an integration of PrEP and the EMR. That is, I think, the only challenge we still have…coming to retrieve the files and moving with the files to every delivery point. But if it can be integrated with the EMR, I think it will be easier.”* (Female Clinician, Facility A).

#### 3.4.3 Client‐centredness

Providers prioritised and fast‐tracked PrEP clients during DTP refill visits to ensure they did not queue at the waiting bay:

*“[DTP] is good because once I have tested myself, the pharmacist…stops providing services to other patients, and you're given [your] drugs first. You then leave and go because he has to continue attending to others.”* (Female Client, Age 24, Facility B).

Strategies to reduce delays and movement between clinic rooms included providing separate locations in the pharmacy for PrEP clients to pick refills, retrieving clients’ files before visits, having clients’ navigators escorting clients to clinic rooms and providers moving files to providers in other rooms:

*“Sometimes, it is us [HIV counsellors] to take that file to the clinician…because of prescription. So, you can take the file, then the clinician prescribes in the absence of the client, then you take it to the pharmacy.”* (Female HIV Counsellor, Facility A).

#### 3.4.4 Provider‐centredness

Providers reported improved teamwork and flexibility in PrEP delivery since most HIV clinic providers were trained in delivering PrEP. Other providers helped with PrEP delivery tasks when the assigned providers had a high workload. In one clinic, clinical providers assisted pharmacy providers in PrEP dispensing or filling clinical encounter forms during high‐volume clinic days:

*“When it comes to a clinic day…we have more than ten [PrEP] clients, with clients living with HIV being very few. So, to try and decongest the pharmacy, the clinician fills the clinical encounter form, then the client will just land to the pharmacy to get the drugs only.”* (Female HRIO, Facility A).

#### 3.4.5 Client learning‐centredness

DTP refill visits empowered clients through active involvement in their HIV prevention actions, particularly through performing HIVST, and from the adherence counselling provided:

*“First, the provider guided me on how I would do it [HIVST]; the second time, they asked me to do it on my own. I felt it was good. You know, at times, you may be exposed…Like I said, condom‐burst before three months are over; you can go to the chemist and buy the self‐testing kit and do the test by yourself at home.”* (Female Client, Age 37, Facility A).

#### 3.4.6 Compatibility with the clinic environment

DTP refill visits were perceived to be easy to adopt and implement as they easily fit in and did not require much change to existing clinic operations and infrastructure. DTP refill visits also addressed previously experienced service delivery challenges in both clinics, particularly poor clinic flow, time inefficiencies for clients and providers, and HIV clinic staff shortages:

*“[DTP] fits well; it doesn't affect how we carry out other services…our daily routine, and it fits well…We only spend extra minutes with the client of which the client saves those minutes by not going to see other healthcare providers, so it really doesn't affect our daily routine.”* (Male Pharmacist, Facility B).

#### 3.4.7 Relative priority and mission alignment

DTP refill visits facilitated the prioritisation of PrEP services within HIV clinics and contributed to the achievement of facility HIV prevention targets. Providers felt PrEP delivery was an important part of their duties and that DTP refill visits complemented their roles. However, HIV counsellors were concerned about the loss of previous roles that involved more interaction with PrEP clients:

*“There were some issues with some HIV counsellors who thought they were not doing their work since they were not serving the PrEP clients the way they were supposed to…So, we were like, “Why are we not doing the work we ought to have been doing?” … [But] we came to understand that it was all for the benefit of our clients.”* (Female HIV Counsellor, Facility A).

#### 3.4.8 Access to knowledge and information

Providers acquired knowledge through training provided on DTP refill visits and PrEP delivery before and during the implementation of DTP refill visits, which enhanced their competence. Continuous on‐the‐job training and supportive supervision by project staff enhanced providers‘ skills and confidence and facilitated ownership of the intervention. This influenced providers’ willingness to implement DTP refill visits and was perceived as key for sustainability:

*“I don't think there is any worry since we have been given refreshers, a lot of information, we have been well oriented, so even if they exit, we understand there is the DTP model, and other providers understand as well, so in case they are called, the client will be attended to even if [client's navigator] is not there.”* (Female HIV Counsellor, Facility B).

### 3.5 Individuals domain

#### 3.5.1 Clients’ needs

##### 3.5.1.1 Reduced opportunity costs

DTP refill visits were perceived to reduce clients’ opportunity costs, including foregoing important activities, long travel distances, high travel costs and getting permission from work to attend clinic visits. This resulted in less disruption of clients’ schedules and reduced concerns about missing work and income among clients. There were fewer concerns of opportunity costs among older male and female clients (≥ 45 years). Younger male and female clients (≤ 40 years) reported reduced risk of losing jobs during DTP refill visits, or exposure to social harm among younger females (≤ 30 years) – due to fewer delays at the clinics, which motivated PrEP continuation:

*“Because the time I spend here at the clinic is little, when the scheduled day for the refill reaches, I feel I have the motivation for coming to pick the drugs because I know I will not waste time. I know I will come then go to my other work.”* (Female Client, Age 40, Facility A).

#### 3.5.2 Provider capacity

Providers were comfortable implementing DTP refill visits, attributed to adequate training, continuous learning opportunities, and ease of learning and implementation. However, PrEP knowledge gaps initially affected confidence among pharmacy providers who were concerned about their readiness to deliver DTP refill visits. Providers later accepted DTP refill visits after knowledge gaps were addressed through training and after observing its benefits to clients:

*“I think the pharmacy was where the problem was…It was said that there was a lot of workload, and they [pharmacy providers] might not be able to fill out the forms. They felt it should be done elsewhere; they should only be left to dispense the drugs.”* (Female HIV Counselling Team Lead, Facility A).

#### 3.5.3 Client and provider motivation

Clients and providers expressed confidence and future willingness to participate in DTP refill visits. Providers were motivated to continue the intervention since they recognised that the changes to client flow were effective, worked well in their clinics, and benefited both clients and providers. Providers believed that DTP refill visits would improve future PrEP uptake and retention:

*“We believe the [PrEP] uptake will be a bit higher because most of the clients were defaulting because they were coming here, they line up, they go through the process over again, and again they come here. Now that it is direct‐to‐pharmacy, I think the uptake will be a bit higher.”* (Male HIV Counsellor, Facility A).

## 4 DISCUSSION

In this project implemented in an existing PrEP programme, DTP PrEP refill visits with HIVST were highly acceptable as a differentiated PrEP delivery model for PrEP clients and providers. The high enthusiasm was attributed to perceived higher service efficiency, quality, and satisfaction related to shorter waiting times and improved client flow. These findings are consistent with the quantitative results of this study population, which showed a high acceptability and satisfaction with DTP refill visits among PrEP clients [31]. In our quantitative data, more than a three‐fold reduction in waiting time and higher PrEP continuations and adherence were reported over the 6‐month follow‐up period in the two intervention clinics compared to the control clinics. Our qualitative findings provide insights to explain the high acceptability of DTP refill visits.

Among clients, high acceptability was attributed to good service quality and satisfaction related to comfort and convenience of short waiting times, improved privacy, reduced stigma and opportunity costs, which increased the motivation for PrEP continuation. Among providers, improved client flow, reduced burden and improved capacity were key facilitators. These findings concur with previous studies that have highlighted strong preference among clients and providers for DSD interventions that provide convenience and reduce clients’ opportunity costs, HIV‐clinic‐associated stigma, provider workload and clinic congestion [40, 41, 42, 43]. DSD interventions within HIV treatment have reported similar outcomes of improved service quality and reduced clients’ opportunity costs, which are major barriers to HIV service access in SSA [44, 45]. The WHO‐endorsed DSD HIV care approaches focus on improving the quality of care through health system efficiency and client‐centredness [46]. Studies in Eswatini, South Africa and Malawi have reported high satisfaction among HIV care clients and providers related to efficiency, convenience and timesaving benefits of DSD models compared to conventional care [47, 48, 49].

A high enthusiasm for implementing DTP PrEP refill visits among providers was attributed to reduced workload among non‐pharmacy cadres, ease of implementation, task‐sharing and compatibility with routine clinic operations. Our findings are consistent with other studies showing the potential of task‐sharing strategies to enhance PrEP delivery with minimal additional resources [50]. We previously showed that the “one‐stop‐shop” PrEP delivery model that involved redesigning workflow and task‐shifting resulted in reduced waiting time, improved privacy for PrEP clients and reduced provider workload within public clinics in western Kenya [29]. Differentiated PrEP delivery strategies within Kenyan public HIV clinics, including longer refills, fast‐tracking clients and task‐shifting, have addressed concerns of long waiting times and HIV clinic stigma, with positive impacts on PrEP uptake and continuation [30, 51]. Pharmacy provider‐led PrEP delivery has been shown to be acceptable in community pharmacy‐based PrEP delivery models in Kenya, South Africa and the United States [52]. In Kenya and Malawi, HIV care clients have reported a preference for individual facility‐based DSD models delivered by pharmacy providers, indicating a potential acceptability of individual facility‐based pharmacy PrEP delivery [53, 54].

The main concerns of DTP refill visits included potential missed opportunities for counselling and clinical assessments among clients and increased workload among pharmacy providers. The changes in client flow were perceived to result in fewer opportunities for counselling, particularly among clients in HIV‐serodifferent relationships, who may need regular counselling [55]. Clients were given an opportunity to request to see a clinician or HIV counsellor if they needed support. However, older male clients may have found it challenging to approach providers compared to younger female clients. Hence, regular reinforcement of the message regarding the availability of counselling may be beneficial for older clients. Counselling is an essential component of PrEP care, and studies have reported concerns of perceived reduced quality of services when clients feel they have less time with clinicians [42, 51]. Proactive screening and innovative counselling strategies may be necessary for clients who need additional support during PrEP refill visits [42, 56]. Client‐centred counselling strategies, focused on improving knowledge, motivation and self‐efficacy, have been shown to result in increased PrEP effective use among vulnerable populations such as AGYW in South Africa and Black women in the United States [57, 58, 59].

Increased pharmacy provider burden and other feasibility concerns were addressed through clinic‐level modifications, with clinicians assisting pharmacy providers in PrEP dispensing. Facility‐based DSD HIV care interventions have been effective in addressing staffing challenges in public HIV clinics, yielding potential cost‐saving benefits through task‐shifting and sharing strategies [60, 61, 62, 63]. An increased workload burden has been reported among some provider cadres implementing task‐shifted DSD HIV care interventions [29, 64]. In Burkina Faso and Eswatini, varying workload concerns were reported, attributed to the additional administrative tasks of filling clinical forms [47, 64]. This is consistent with our findings where workload burden was related to additional documentation roles and staffing shortage. However, the perceived benefits to clients outweighed workload concerns [29]. Continuous monitoring and adaptations to address workload concerns may be critical. In our setting, the task‐sharing modification—although unintended, continuous provider training, and the observed benefits to clients and providers, contributed to the acceptability of the DTP model, indicating a high likelihood for its sustainability.

We found a high willingness among clients and providers to continue participating in DTP PrEP refill visits. The DTP model also aligned with multi‐level factors critical for the sustainability of healthcare innovations. These included observable client outcomes; adaptability, low complexity and burden; client and provider satisfaction and belief in the intervention; alignment with clinic priorities and culture; staff training, ownership and capacity to implement the intervention [65, 66, 67]. However, future implementation and scalability of the DTP model may largely depend on resource availability since shortages of commodities such as HIVST kits and PrEP supplies experienced during the COVID‐19 pandemic, and inadequate pharmacy staffing, EMR systems and space, may affect sustained implementation. Continuous provider training and supervision, community engagement, facility leadership support, integration in PrEP service guidelines, and continuous monitoring and evaluation will also be crucial for sustainability [68, 69].

Our study had strengths and limitations. First, our study population was largely comprised of individuals in HIV‐serodifferent relationships; hence, the perspectives presented could have been different from those of other populations. Second, we focused on the experiences of clients and providers during the implementation of DTP refill visits, which limited the comparison of acceptability before and after implementation. Third, participants’ selection was based on willingness to consent for study participation and experience with DTP, which may have restricted a balanced representation of clients’ sex and provider cadres. We also interviewed a small sample of PrEP clients and providers within two public HIV clinics, limiting the generalisability of our findings. Our study had the strength of exploring in‐depth perspectives of PrEP providers and users, providing an adequate representation of real‐world experiences with DTP refill visits.

## 5 CONCLUSIONS

In this study aimed at improving the efficiency of PrEP delivery in a real‐world setting within public HIV clinics, DTP refill visits demonstrated high acceptability, feasibility and sustainability among PrEP clients and providers. DTP PrEP refill visits achieved the key outcome of improved service efficiency, yielding positive impacts for clients, providers and health systems. Context‐specific adaptations and scale‐up of the intervention could improve the efficiency of PrEP delivery within public HIV clinics in Kenya and similar settings. Future studies could target other priority sub‐populations of PrEP users and offer tailored, differentiated care strategies, including alternative PrEP delivery locations, provider training models and task‐sharing among different provider cadres.

## COMPETING INTERESTS

The authors declare no competing interests.

## AUTHORS’ CONTRIBUTIONS

Study conceptualisation and funding acquisition: KM. Qualitative study design: KM and KN. Data collection: EO, VO and NW. Project administration: KM, KN, LE, WW, CK and NM. Data analysis: EO, VO and NW. Writing—original draft: EO. All authors reviewed and edited the manuscript and approved the final version.

## FUNDING

This study was supported by funding from the National Institute of Mental Health of the US National Institutes of Health (grants R00MH118134 and R01MH123267).

## Data Availability

De‐identified transcripts will be available upon author request and under appropriate data‐sharing agreements to those who provide a methodologically sound proposal by contacting the International Clinical Research Center at the University of Washington (Email: icrc@uw.edu).
